# 5-hydroxymethylcytosine and gene activity in mouse intestinal differentiation

**DOI:** 10.1038/s41598-019-57214-z

**Published:** 2020-01-17

**Authors:** Santiago Uribe-Lewis, Thomas Carroll, Suraj Menon, Anna Nicholson, Piotr J. Manasterski, Douglas J. Winton, Simon J. A. Buczacki, Adele Murrell

**Affiliations:** 1 Cancer Research UK Cambridge Institute, University of Cambridge, Robinson Way, Cambridge, CB2 0RE UK; 2 Bioinformatics Resource Center, The Rockefeller University New York, New York, NY 10065 USA; 3 Wellcome MRC Cambridge Stem Cell Institute, University of Cambridge, Addenbrookes Biomedical Campus, Cambridge, CB2 0AF UK; 4 Department of Biology and Biochemistry, University of Bath, Claverton Down, Bath, BA2 7AY UK

**Keywords:** Differentiation, DNA methylation

## Abstract

Cytosine hydroxymethylation (5hmC) in mammalian DNA is the product of oxidation of methylated cytosines (5mC) by Ten-Eleven-Translocation (TET) enzymes. While it has been shown that the TETs influence 5mC metabolism, pluripotency and differentiation during early embryonic development, the functional relationship between gene expression and 5hmC in adult (somatic) stem cell differentiation is still unknown. Here we report that 5hmC levels undergo highly dynamic changes during adult stem cell differentiation from intestinal progenitors to differentiated intestinal epithelium. We profiled 5hmC and gene activity in purified mouse intestinal progenitors and differentiated progeny to identify 43425 differentially hydroxymethylated regions and 5325 differentially expressed genes. These differentially marked regions showed both losses and gains of 5hmC after differentiation, despite lower global levels of 5hmC in progenitor cells. In progenitors, 5hmC did not correlate with gene transcript levels, however, upon differentiation the global increase in 5hmC content showed an overall positive correlation with gene expression level as well as prominent associations with histone modifications that typify active genes and enhancer elements. Our data support a gene regulatory role for 5hmC that is predominant over its role in controlling DNA methylation states.

## Introduction

Intestinal epithelium is produced when stem progenitors at the base of intestinal crypts exit from their proliferative state and differentiate^1^. Since stem progenitors and differentiated progeny have identical genomes, their differential gene expression states are achieved through genome modulation by epigenetic factors. The precise nature of the epigenetic mechanisms involved in intestinal homeostasis are still poorly understood as are the role of the epigenetic modifications and the complexes that define them^2–4^.

Cytosine hydroxymethylation (5hmC) has been identified as the oxidation product of methylated cytosines (5mC) by the Ten-Eleven-Translocation enzymes (TETs)^5,6^. Subsequently, 5hmC and TETs (TET1, TET2 and TET3) have been profiled in many pluripotent and somatic cell types^7–15^ as well as neoplasias^16–25^. Consistent with a role in epigenetic reprogramming, the absence of TETs disrupts DNA methylation patterns^26^, hampers embryonic development^27,28^, impairs somatic cell transfer^29^ and promotes neoplasia^30,31^. In addition to being an intermediate in active DNA demethylation^32^, 5hmC has been shown to be a predominantly stable DNA modification^13^. Within the genome, 5hmC is located at transcriptionally active genes and regulatory elements^11,14^ and chromatin associated complexes^33–36^.

The proliferating gut crypt progenitors, from which tumours can arise^37^, show reduced levels of 5hmC relative to the differentiated epithelium^25,38–40^. In this study we have mapped gene expression and 5hmC in purified stem progenitors and differentiated epithelium of the adult mouse intestine to identify which 5hmC-marked genes play a role in intestinal differentiation.

## Results and Discussion

**Figure 1:**
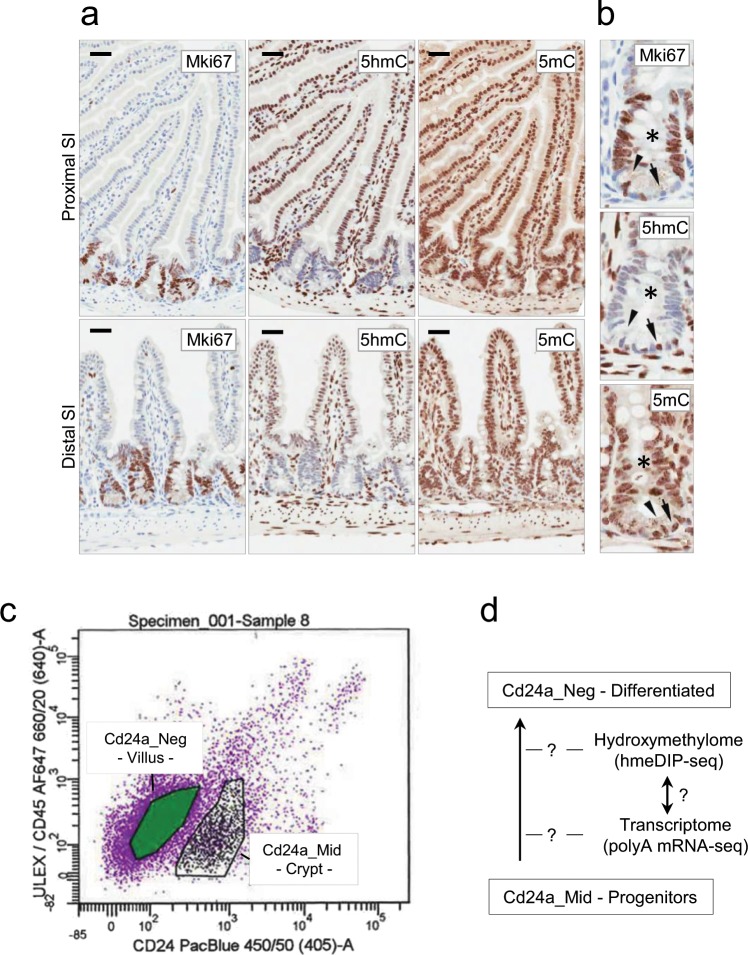
5hmC is low in the proliferating gut crypt progenitors and increased in the differentiated villus epithelium of the mouse small intestine (SI). (**a**) Immunohistochemistry for Mki67, 5hmC and 5mC in the mouse proximal and distal small intestine (SI). Horizontal bars = 20 um. (**b**) Detail for the crypts in the distal SI. The asterisk indicates the transient amplifying zone positive for Mki67 and low for 5hmC. The negative correlation between Mki67 and 5hmC is also observed for the crypt base columnar cells (arrowheads) and the Paneth cells (arrows). Methylation levels do not appear to differ between these cell populations. A negative correlation between Mki67 and 5hmC was also observed in the colon and ApcMin small intestinal adenomas (Supplementary Figure S1). (**c**) Flow cytometry dot plot for the Cd24a_Mid and Cd24a_Neg populations. Ulex-lectin was used to deplete differentiated Paneth and Goblet cells and Cd45 to remove hematopoietic cells. (**d**) The genomic profiles of 5hmC and how they may correlate with gene activity upon gut differentiation were assessed by profiling expresssion (RNA-seq) and 5hmC (hmeDIP-seq) of the purified crypt stem progenitors (Cd24a_Mid) and differentiated villi (Cd24a_Neg).

Initially we confirmed the global levels of 5hmC by immunohistochemistry and the correlation with proliferation by staining with the Mki67 marker (Fig. 1a,b). Mki67 positive crypts in the proximal and distal small intestine (SI) showed lower levels of 5hmC relative to the Mki67 negative villi and crypt Paneth cells as well as cells within the stroma (Fig. 1a,b). The negative correlation between 5hmC and Mki67 was also observed in mouse colon and ApcMin^41^ SI adenomas (Supplementary Fig. S1). Staining for 5mC showed equal levels in crypts, villi and stroma. These results confirm previous observations in mouse and human normal and neoplastic colon^17,25^.

We then used the Cd24a cell surface marker and flow cytometry to purify stem progenitors (Cd24a_Mid) and differentiated cells (Cd24a_Neg) (Fig. 1c). The Cd24a_Mid (low) expressors have previously been shown to carry pluripotency potential^42^. Here we further purified Cd24a_Mid and Neg populations by including Ulex-lectin and Cd45 to remove Paneth/Goblet cells and hematopoietic cells respectively as previously described^43^. Aliquots of Cd24a_Mid (progenitors) and Cd24a_Neg (differentiated progeny) populations were used to isolate total RNA to measure gene expression by poly-A mRNA-seq and genomic DNA to map 5hmC by hmeDIP-seq (Fig. 1d).

**Figure 2:**
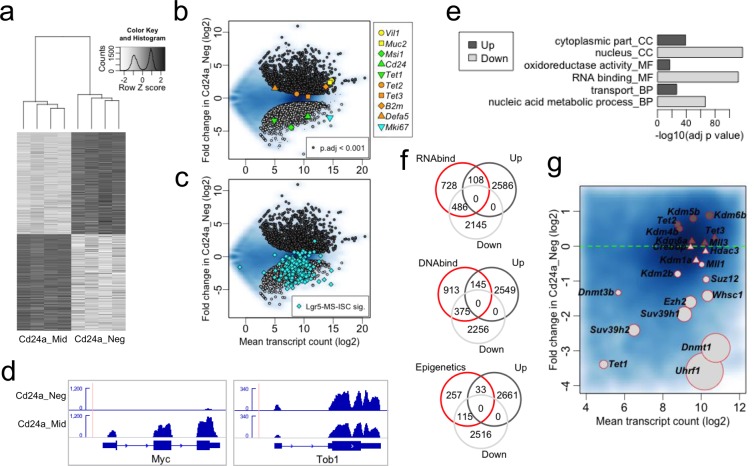
RNA-seq. The intestinal stem cell signature and behaviour of epigenetic factors. (**a**) Cluster heatmap for loci with a statistically significant change (adjusted p value < 0.001) in gene expression of the four samples analysed. (**b**) MA scatter plot for expression change in Cd24a_Neg relative to Cd24a_Mid cells. Blue background represents all genes overlaid by significantly upregulated (dark grey) and downregulated (light grey) genes. Also overlaid are differentiation-markers *Villin1* (*Vil1*) and *Mucin2* (*Muc2*), stem cell marker *Musashi1* (*Msi1*) and *Cd24a, Tet1–3* hydroxylases*, B2m* microglobulin ‘housekeeper’, Paneth-cell marker *Defensin alpha 5* (*Defa5)* and proliferation marker *Mki67*. (**c**) Gene expression change in Cd24a populations compared to the intestinal stem cell (ISC) signature derived from mass spectrometry analysis of Lgr5-GFP mice (Lgr5-MS-ISC, Muñoz *et al*. – listed in Supplementary Table S3). (**d**) Browser shot for RNA-seq reads levels in *Myelocytomatosis oncogene - Myc* (−3.4 log2 fold, p.adj = 1.4e-48) and *Transducer of ErbB-2.1 - Tob1* (1.3 log2 fold, p.adj = 5.3e-11). (**e**) Top gene ontology categories unique for Up or Down regulated genes (See Supplementary Fig. 4 for extended display of GO categories). (**f**) Venn plots for RNA and DNA binding factors as well as collated epigenetic factors (listed in Supplementary Tables S6-S8). (**g**) MA scatter plot for expression change in Cd24a_Neg relative to Cd24a_Mid cells. Blue background represents all genes overlaid by selected epigenetic factors. Circle sizes are the inverse log of the adjusted p value (smaller p-values produce larger circles). As reference, padj for *Uhrf1* is 6.4e-68 whereas *Mll1* is at 3.9e-02. Triangles indicate a p value > 0.05.

RNA-seq showed 2694 and 2631 genes that were significantly up and downregulated respectively with an adjusted p value (p.adj) < 0.001 in differentiated cells (Fig. 2 and Supplementary Tables S1 and S2). Unsupervised hierarchical clustering clearly separated the two cell types (Fig. 2a) and expression changes included gain of differentiation markers *Villin1* (*Vil1*) and *Mucin2* (*Muc2*) and loss of stem cell marker *Musashi 1* (*Msi1*) (Fig. 2b). In agreement with reduced protein expression, *Cd24a* and *Mki67* transcripts were strongly reduced in the Cd24_Neg cells (Fig. 2b). The Paneth cell marker *Defensin alpha 5* (*Defa5*) was not significantly increased in Cd24a_Negs (Fig. 2b) as expected from Paneth cell depletion with Ulex-lectin. These loci were validated by qRT-PCR (Supplementary Fig. S2). High concordance was also observed for significantly downregulated genes in Cd24a_Neg cells, i.e. stem progenitor specific, with loci recently shown to mark intestinal stem cells by mass spectrometry and the Lgr5 stem cell factor^44^ (Fig. 2c and Supplementary Table S3). Moreover, marked reduction of *Myelocytomatosis oncogene* (*Myc*) and increased levels of *Transducer of ErbB-2.1* (*Tob1*) (Fig. 2d) support Wnt pathway inhibition and Bmp pathway activation, respectively, that characterizes differentiation of the adult intestine^1^. These results confirm that the purification strategy can robustly separate stem progenitors from differentiated cells, and provide a powerful resource for future analyses.

Remarkably, changes in *Tets* transcripts levels were moderate and did not always mirror the increase in global levels of 5hmC upon differentiation, similar to our pervious observations for reduced 5hmC in human colon neoplasia^25^. *Tet1* levels were low in Cd24a_Mid progenitors and went down with differentiation, *Tet2* was reasonably abundant in progenitors with a mild increase in differentiated progeny and *Tet3* the most abundant of the *Tets* with levels maintained in the Cd24a_Neg differentiated cells (Fig. 2 and Supplementary Fig. S2). Our results in this regard appear to differ from other published studies^39,40^. Although Kim *et al*.^39^ also observed a marked reduction of *Tet1* upon differentiation, they showed that *Tet1* was the most abundant of *Tets*. This difference may be due to the alternative stem cell purification approach. Chapman *et al*. showed that TET1 expression increases upon *in vitro* colonocyte differentiation^40^. This disagrees with our study and that of Kim et al. and may be due to species-specific differences or cell culture effects.

We additionally observed no alternative exon usage for *Tet2* or *Tet3* between Cd24a_Mid and Cd24a_Neg cells (Supplementary Fig. S3) suggesting that oxygenase activity in the progenitors and differentiated cells might be regulated by post-transcriptional events^45–49^.

Goseq^50^ analyses of the differentially expressed genes in progeny and pluripotent cells (Supplementary Tables S4 and S5) showed that upregulated genes enriched for gene ontology (GO) categories involved in cellular metabolic functions localized to the cytoplasm whereas downregulated loci enriched for RNA binding factors and nucleic acid metabolic processes within the nucleus (Fig. 2e and Supplementary Fig. S4). These GO profiles are consistent with enrichment of enterocytes in the Cd24a_Negs and enrichment of proliferating stem progenitors in the Cd24a_Mid cells. The RNA binders (GO:0003723) include the stem cell marker MSI1 but also methyl-CpG binding factor MECP2, that also directly interacts with DNA^51^. *Mecp2* was significantly downregulated in Cd24a_Neg cells (p.adj = 2.4e-03, Supplementary Table S6) but with overall low levels as recently described^52^. The DNA binding category (GO:0003677) was also significantly enriched in genes downregulated in Cd24_Neg cells (p.adj = 1.5e-16, Supplementary Table S7 and Fig. 2f).

To further focus the analysis on epigenetic factors that establish, recognize or erase epigenetic modifications, many of which are not classified as nucleic acid binders, we collated epigenetic modifiers and interactors (Supplementary Table S8)^53,54^. Again, we observed a strong bias towards downregulation of these loci (Fig. 2f bottom). Notably, key factors involved in methylation of DNA (*Uhrf1, Dnmt1, Dnmt3b*) and histones (*Suv39h* – H3K9*, Ezh2* – H3K27*, Suz12* – H3K27*, Whsc1* – H3K36*, Mll1* – H3K4) were downregulated whereas most factors involved in demethylation of DNA and histones were either moderately upregulated (*Tet2, Tet3* – 5mC, *Kdm4b* – H3K36*, Kdm5b* – H3K4*, Kdm6b –* H3K27) or their levels maintained (*Kdm1a –* H3K4*, Kdm6a –* H3K27)(Fig. 2g). Two exceptions were *Tet1* that was downregulated from an already low level in progenitors and mild downregulation of *Kdm2b*, a histone H3K4 and K36 demethylase that binds CpG islands of early lineage genes in mouse embryonic stem cells^55,56^. *Crebbp* and *Hdac3*, encoding for enzymes that acetylate and deacetylate histone H3 lysine 27 respectively^57^, were abundant and maintained throughout differentiation. This may indicate that levels of H3K27ac, a mark of enhancer elements^58^, are maintained throughout differentiation. Although post-transcriptional events influencing the stability or activity of epigenetic factors cannot be discerned by RNA-seq, these results show that intestinal differentiation involves a complex balance in the levels of a considerable number of epigenetic factors (Supplementary Table S8).

**Figure 3:**
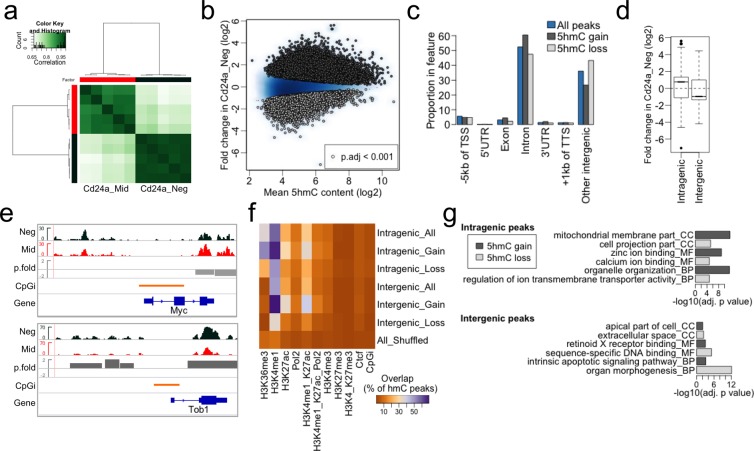
Dynamic behaviour of 5hmC across the genome. (**a**) Correlation heatmap using affinity values (reads in peaks) from ‘DiffBind’. Clustering of the individual samples (four of which are paired to RNA-seq data) was obtained as well as an increased signal for the Cd24a_Neg cells consistent with the increased global level observed by IHC (Fig. 1). (**b**) MA scatter plot for the fold change in 5hmC content in Cd24a_Neg cells relative to Cd24a_Mid. 21858 peaks showed gain (dark grey) and 21567 loss (light grey) of 5hmC with an adjusted p value < 0.001 for the change across the sample set. The blue background is the density scatter plot for all peaks identified (n = 97309). (**c**) 5hmC contents in genomic features (PAVIS analysis). (**d**) Boxplot for the fold change in 5hmC content at intragenic and intergenic peaks. (**e**) IGV browser shot of 5hmC enrichment profiles for *Myc* and *Tob1* in Cd24a_Neg and Cd24a_Mid cells. The bigWig files represent a merge of reads from each Cd24a population (n = 4). P.fold is the peak fold change (log2) in hmeDIP-seq read content (normalized to inputs). The bar heights are proportional to the magnitude of loss (in light grey) or gain (in dark grey) in 5hmC content. Shown are peaks with an adjusted p value < 0.001. Orange bars are the CpG islands from the UCSC annotation. The window size for *Myc* and *Tob1* is 15 kb. Please see Supplementary Figure S5 for an extended 100 kb window). (**f**) Heatmap summarising the overlap between 5hmC peaks and ChIP-seq peaks for histone modifications, POL2 and CTCF from mouse whole small intestine (ENCODE). (**g**) Gene ontology analysis for genes associated with 5hmC change inside (intragenic) or outside (intergenic) the gene body. Shown are categories that are unique to each condition (Supplementary Fig. S8 shows a more extended display of GO categories).

Next, we profiled 5hmC by hmeDIP-seq in four samples matched to those used in RNA-seq plus one additional pair. Cluster analysis using affinity values (reads in peaks) by DiffBind^59^ showed a clear separation between the Cd24a_Mid and Neg samples together with increased affinity in the Cd24a_Neg cells (Fig. 3a), in line with the global increase of 5hmC levels upon differentiation. Notably however, when we used an adjusted p value of 0.001, we obtained a roughly equal number of peaks that gained or lost 5hmC (21858 and 21567 peaks respectively out of 97309 peaks identified) (Fig. 3b, Supplementary Tables S9 and S10). Peak annotation and visualization (PAVIS) analysis^60^ showed that ~60% of peaks were intragenic, mainly within introns, but statistically significant enrichments in exons (p < 1.00e-200) and 3′UTRs (p < 1.00e-15) were obtained. The remaining ~40% of 5hmC peaks were intergenic of which ~5% were within 5 kb of the transcription start sites – TSS) (Fig. 3c). Gain of 5hmC during differentiation mainly occurred inside genes, both at introns and within exons, with 5hmC loss more frequent at distant intergenic sites (>5 kb upstream of TSS or >1 kb downstream of TTS) (Fig. 3c,d). *Myc* had loss and *Tob1* gain of 5hmC within the gene body and upstream intergenic regions (Fig. 3e and Supplementary Fig. S5), showing that 5hmC change and gene expression change (Fig. 2d) at these loci were positively correlated.

Immunoprecipitation sequencing for DNA modifications has been reported to be intrinsically enriched for short tandem repeats (STRs) due to non-specific antibody binding affinity^61^. An IgG-only control was not included in our experimental design. The DNA pull-down conditions are identical for Cd24a_Mid and Neg cells and thus it would be assumed that non-specific sequences are unlikely to be statistically significantly different between the two conditions. With this in mind we conducted a motif discovery analysis (MEME-ChIP^62^) to assess sequence contents in peaks that gained or lost 5hmC in progeny. Given the peak size and p value cut-off used to select peaks with 5hmC change (Supplementary Fig. S6) the analysis showed CpG-dinucleotide-containing motifs emerged from loci that gained 5hmC and CA repeat sequences predominant at regions with 5hmC loss. This could suggest that immunoprecipitation for 5hmC enriches for STRs when 5hmC is in low abundance. However, intersection of 5hmC peak intervals with (CA)n simple repeat intervals showed the repeats are more abundant in peaks with 5hmC loss (8.3% in loss versus 3.7% in gain of all 5hmC peaks identified; Supplementary Fig. S6). CA repeat sequences can occur adjacent to CpG rich sequences and have been shown to play a role in regulating dynamic changes in 5hmC and DNA methylation during differentiation^63^. Moreover, the contribution of non-specific antibody activity (measured as abundance of STRs) is also relatively low. We therefore did not filter our data to remove CA repeats.

To integrate our 5hmC maps with epigenomic features involved in gene regulation we analysed the overlap of 5hmC peaks with genomic profiles of histone modifications. Since our flow sort material was insufficient to conduct ChIP measurements we used whole small intestine ENCODE ChIP-seq data. In this manner 5hmC dynamics would be measured within ‘static’ histone modification intervals to assess an overall ‘interaction’. We observed an overlap with histone H3 lysine 36 trimethylation (H3K36me3) peaks, that marks actively transcribed genes^57^ primarily for intragenic peaks that gain 5hmC (Fig. 3f). A much greater overlap was found between H3K4me1 peaks and those that gain 5hmC at intergenic sites, and a considerable overlap was also observed with gain of 5hmC in intragenic regions (Fig. 3f). H3K27ac and bivalent H3K4me1/H3K27ac sites also often overlapped with 5hmC peaks, most frequently with intragenic and intergenic sites that gained 5hmC. These results are in good agreement with enrichment of 5hmC at poised and active enhancers recently described in mouse embryonic and somatic cells^10,11^.

Low frequency overlaps with H3K4me3 peaks were observed (Fig. 3f). This was expected given that H3K4me3 locates mostly to transcriptional start sites (TSS) and promoter CpG islands where 5hmC is normally absent^25^. H3K27me3 and bivalent H3K4/K27me3 sites also showed a minimal overlap with 5hmC. However, genes with promoters marked by H3K4me3 within 5 kb of the TSS (n = 11104) were for the most part highly expressed in Cd24a_Mid and Cd24a_Neg cells and more frequently gained 5hmC (n = 3130, ~28%), albeit a considerable number lost 5hmC (n = 1477, ~13%) at the cut-off used for significant changes in 5hmC (p.adj < 0.001) (Supplementary Fig. S7). On the other hand, genes with bivalent H3K4/K27me3 promoters (n = 3384 promoters) showed constitutively lower levels of gene activity and more frequently loss of intragenic 5hmC (~23% gain against ~10% loss of intragenic 5hmC) in the differentiated progeny (Supplementary Fig. S7).

Only a small number of 5hmC peaks were found to overlap with CTCF, a chromatin associated factor involved in long-range genetic interactions^64^ (Fig. 3f). ENCODE ChIP-seq profiled whole intestine, precluding resolution of stem cell specific signatures such as histone bivalency at promoters and enhancers. Nevertheless, these results identify negative and positive associations between 5hmC changes and key histone modifications.

GO analysis for genes with significant 5hmC intragenic changes (Supplementary Tables S11 and S12) showed enrichments of GO categories associated with cellular metabolism and cell-cell interaction (Fig. 3g and Supplementary Fig. S8) akin with GO category enrichments associated with upregulated genes. However, GO category enrichments driven by these intragenic 5hmC changes were similar for genes that gained or lost 5hmC. Intergenic 5hmC peaks were assigned to the nearest gene and included the proximal promoters. We observed enrichments for GO categories associated with cell signaling, DNA template processes and organ morphogenesis (Fig. 3g and Supplementary Fig. S8) – again irrespective of the direction in 5hmC change (Supplementary Fig. S8 and Supplementary Tables S13 and S14) and in this instance akin with GO category enrichments associated with downregulated genes (Supplementary Fig. S4). These GO analyses would suggest that stemness in mouse intestine may be primarily controlled by intergenic regulatory elements and suggest that while gene activation or silencing is associated with changes in 5hmC, these changes are dependent on the genomic context and not strictly directional.

**Figure 4:**
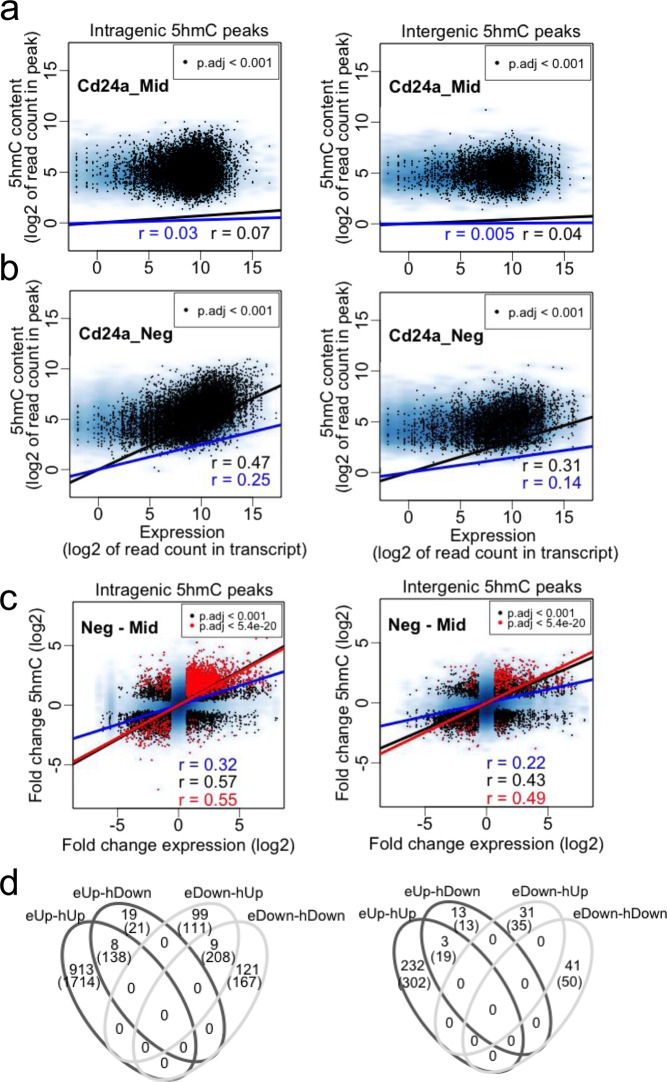
Gene expression and 5hmC in progenitors (Cd24a_Mid) and progeny (Cd24a_Neg). (**a**) Correlation between expression levels and 5hmC content at intragenic or intergenic peaks in the Cd24a_Mid progenitors. Blue are all loci, black are loci with an adjusted p value < 0.001 for expression and 5hmC change between progenitors and progeny. (**b**) As in (**a**) but for the Cd24a_Neg progeny. (**c**) Correlation between the fold change in expression and the fold change in 5hmC content from progenitors to progeny at intragenic or intergenic peaks. The blue background represents all loci, the black overlay are loci with an adjusted p-value < 0.001 for the change in expression and 5hmC content. The red overlay are loci with and adusted p value < 5.4e-20 for 5hmC change (this is the mean of adjusted p values for loci with an absolute log2 fold change > 2). (**d**) Venn diagram intersecting gene symbols in the four populations at the stringent p value showing that genes (open numbers) that gain or lose expression (eUp or eDown respectively) can contain peaks (n in brackets) with only gain, only loss or gain and loss of 5hmC (hUp, hDown or hUpDown respectively).

We therefore took a closer look at the association between gene expression and 5hmC genomic contexts. In the progenitors (Cd24a_Mid) we observed no correlation between expression levels and 5hmC for both intragenic and intergenic 5hmC peaks (Fig. 4a). The correlation coefficient became positive when expression level and 5hmC content were compared in progeny (Cd24a_Neg), but more so for loci with significant changes in expression and 5hmC between progenitors and progeny (Fig. 4b). This analysis indicated that the gene expression programme of proliferating progenitors (stem cells) does not require high levels of 5hmC and suggested a stronger association between expression change and 5hmC change upon lineage inductions.

The correlation coefficients rose further when the fold changes in expression were compared to the fold changes in 5hmC (Fig. 4c). For intragenic 5hmC peaks, comparison of significant fold changes in expression estimates *per gene* (this is the sum of reads for all annotated transcripts of a gene), with significant fold changes in 5hmC contents of *all intragenic peaks per gene* showed an overall positive but moderate correlation (r = 0.57 for p.adj < 0.001 in expression and 5hmC change) (Fig. 4c). In agreement with GO analysis for 5hmC enrichments described above, upregulated and downregulated expression could be accompanied by either gain or loss of 5hmC. Increasing the significance cutoff for 5hmC changes to an adjusted p value of 5.4e-20 (this is the mean of adjusted p values for loci with an absolute log2 fold change greater than 2) did not greatly affect the correlation coefficient (r = 0.55) (Fig. 4c). It is worth highlighting here that the correlation coefficients may be underestimated given that poly-A enrichment depletes intronic transcripts where 5hmC is considerably enriched (Fig. 3c). Nevertheless, at the stringent p value the large majority of genes showed a positive correlation between expression change and intragenic 5hmC change, i.e. expression Up with 5hmC Up (eUp-hUp) or expression Down with 5hmC Down (eDown-hDown) (1034 out of 1169 genes, 88.5%, Fig. 4d). Ten percent of genes (118 out of 1169) showed a negative correlation between expression and 5hmC change (i.e. eUp-hDown and eDown-hUp), and a small number of genes (~1.5%, 17 out of 1169) showed expression change in one direction with 5hmC changes in both directions (i.e. eUp-hUpDown and eDown-hUpDown) (Fig. 4d).

A similar behaviour was observed for the correlation between gene expression change and 5hmC change at intergenic peaks (Fig. 4c) albeit that the number of significant changes in 5hmC was smaller and correlation coefficients weaker. Nevertheless, the proportions of genes with positive, negative and mixed correlations (85%, 14% and 1% respectively, Fig. 4d) were similar to those observed for intragenic 5hmC changes.

Collectively, these results reinforce previous observations of the preferential association of 5hmC with active loci, but also highlight that 5hmC may associate with repressor^65^ or activator^34^ activities. In this regard, two additional observations are relevant. First, genes with very low (no) expression had considerable levels of 5hmC in progenitors, and the 5hmC levels were significantly reduced upon differentiation, whereas genes with undetected 5hmC showed low levels of expression in both progenitors and differentiated cells (Supplementary Fig. S9). This contrasts with active and 5hmC enriched genes in progenitors where the levels of 5hmC and expression are increased in differentiated cells (Supplementary Fig. S9). Second, we noted that higher levels of 5hmC in the proximal promoter associated with a lower level of activity (Supplementary Fig. S10), in agreement with recent reports in mouse and human ES cells^66,67^ and human colon^25^.

These results show that highly repressed genes can contain 5hmC but contrary to active genes 5hmC goes down with differentiation, and that genes lacking 5hmC have constitutively low levels of expression. Thus presence of 5hmC in progenitors and further accumulation in progeny would appear to be necessary for the re-tuning of gene expression states in differentiation of the adult intestinal epithelium. This assumption, that requires experimental confirmation, would be supported by hampered embryonic development and off track lineage commitment of mouse ES cells in the absence of TETs^28^.

## Conclusions

Here we confirm a global increase in 5hmC occurs from the stem progenitors to the differentiated progeny in mouse small intestinal epithelium^17^. Importantly, despite the rise in global levels, we show that 5hmC is highly dynamic with prominent gains and losses across the genome of differentiated progeny. The dynamic behaviour of 5hmC is in stark contrast with the evidence of a remarkably stable methylome during intestinal differentiation^68^. In this context, our results suggest that for the most part 5hmC would not act to control DNA methylation states. Conversely, given the prominent association of 5hmC with histone modifications of active loci and enhancer elements, our results suggest 5hmC may be primarily involved in controlling gene activity. These data provide a valuable resource for future mechanistic insights into the association of DNA modifications and gene activity.

Recent reports have also indicated that broadly permissive chromatin structures typify differentiation of the small intestine and that the phenotypic changes are primarily driven by transcription factor activities^4,69^. These reports question the function of the global changes in epigenetic modifications observed upon differentiation^2,17,38^. We have recently shown that rapidly cycling cells show a delay in the generation of 5hmC, and that once established it is very stable^13^. Our data suggest that this may also hold true for histone modifications, given the inverse correlations between levels of modifiers with levels of the modifications (e.g. downregulation of Ezh2 with a rise in H3K27me3).

Our data highlight pronounced changes in epigenetic factors in mouse small intestinal differentiation. Whether these changes follow or instruct intestinal differentiation, or both, remains largely unknown. However, orchestrated targeting of epigenetic complexes in intestinal neoplasia^70,71^ suggests epigenetic factors would strongly influence the functional outputs of transcription factor activities.

## Materials and Methods

### Animals

Intestinal tissue was obtained from C57BL6/J and ApcMin mice that were housed and bred in the Cancer Research UK Cambridge Institute Biological Resource Unit (CRUK-CI BRU) in compliance with the statutes of the Animals (Scientific Procedures) Act, 1986, UK Home office guidelines and approved by the University of Cambridge Animal Welfare and Ethical Review Body.

### IHC

IHC was conducted in the Histopathology core facility at the CRUK Cambridge Institute. The IHC protocol for Rabbit anti-5hmC polyclonal (Active Motif, 39791), Mouse anti-5mC monoclonal (Diagenode, MAb-006) and Rat anti-Mki67 monoclonal (Dako, M7249) was previously described^13^. Slides were scanned onto an Aperio scanner for analysis. Antibodies go through a strict validation pipeline including a no primary antibody control staining to ensure secondary antibodies do not cross react with the tissue (Supplementary Fig. S11).

### Intestinal epithelium fractionation and flow sort

Mouse intestinal epithelium was obtained by EDTA based fractionation as previously described^43^. Single cell suspensions of the whole small intestinal denuded epithelium were sorted into Cd24a-Mid_Cd45-negative_UEA-1-negative and Cd24a-Negative_CD45-negative_UEA-1-negative populations using Pacific blue conjugated Rat anti-CD24 (Biolegend, M1/69, 5 μL/10^6^ cells), Alexa647 conjugated Rat anti-CD45 (BD Pharmingen, Cat. No. 557683, 1:200) and Atto-488 conjugated UEA-1 (ULEX) (Sigma, 10 μL/10^6^ cells) on a FACSAria (BD Biosciences) flow cytometer.

### RNA-seq

Total RNA was extracted with the miRNeasy kit (Qiagen) following manufacturer instructions. Total RNA from four Cd24a_Mid and four Cd24a_Neg samples were submitted for library preparation by the CI-genomics core facility using the TruSeq RNA Sample Preparation Kit (Illumina). Barcoded samples were sequenced on a single lane of Illumina HiSeq to a depth of more than 200 million paired end (PE) 100-based pair (BP) reads. After demultiplexing, this yielded between 17.7–26.5 million PE reads per sample. These reads were trimmed to 50BP and aligned to mouse transcriptome version NCBIM37.67 using Bowtie version 0.12.8^72^. Gene read counts were then derived using the MMSeq^73^ workflow. Differential gene expression analysis was carried out on these read counts using the Bioconductor package DESeq^74^. DEXSeq package^75^ was used to quantify reads within intervals obtained from Ensembl NCBIM37.67 gtf.

### hmeDIP-seq

Genomic DNA was obtained by phenol chloroform extraction and sonicated with a Bioruptor (Diagenode) to an average fragment size of 500 bp. The 5hmC pulldown was performed as recently described^25^ using protein G magnetic beads (LifeTechnologies) bound with 5hmC rabbit polyclonal antibody (Active Motif, 39791) and 2 micrograms of adapter modified barcoded genomic DNA (TruSeq, Illumina). Illumina sequencing reads were demultiplexed and aligned against the mm9 genome assembly using BWA. Quality metrics of the hmeDIP-seq enrichments were obtained with ChIPQC^76^. DiffBind package^59^ was used to quantitatively compare reads within peak sets obtained with MACS and differential affinity with the edgeR workflow after read counts from input DNA were subtracted. Mean read coverage around TSS was calculated using ‘GenomicRanges’ and ‘Rsamtools’ (Bioconductor); read coverage was normalized per million mapped reads, subtracted from input and mean TSS coverage plotted. Feature Enrichment analysis used the PAVIS online tool^60^. Summary statistics for hmeDIP-seq reads are in Supplementary Table S15).

### Gene ontology

The goseq package^50^ was used for gene ontology analyses of RNA-seq and hmeDIP-seq data.

### MEME-ChIP

Motif analysis of hmeDIP-seq peaks was performed using the online tool with default parameters. The primary sequences within selected peaks were obtained with bedtools.

### CA repeat overlaps

The (CA)n Simple_repeat intervals were extracted from the UCSC RepeatMasker table. Intersection of 5hmC peaks intervals with (CA)n repeat intervals was conducted using bedtools. The (CA)n repeats had to be fully contained within 5hmC peaks (i.e. F = 1).

### qRT-PCR

1 microgram total RNA was treated with 1U DNaseI (Promega 9PMIM610) and cDNA prepared with SuperscriptIII reverse transcriptase (Invitrogen) and random primers. Targets were quantified with 1x Fast Sybr (ABI) and 1x Quantitect assays (Qiagen) or Taqman assays by the delta CT method using *B2m* as normalizer (Supplementary Table S16).

## Supplementary information

Supplementary information

Supplementary information2
