# Supplementary material for: 5-hydroxymethylcytosine and gene activity in mouse intestinal differentiation
Source: Sci Rep. 2020 Jan 17;10:546. doi: 10.1038/s41598-019-57214-z (PMC6969059; doi:10.1038/s41598-019-57214-z)
Supplement: Supplementary file 2 — Supplementary information2 [file 41598_2019_57214_MOESM2_ESM.pdf]

5-hydroxymethylcytosine and gene activity in mouse intestinal differentiation.

Santiago Uribe-Lewis, Thomas Carroll, Suraj Menon, Anna Nicholson, Piotr J Manasterski, Douglas J. Winton, Simon J.A. Buczacki & Adele Murrell*

Supplementary Figures S1-S11

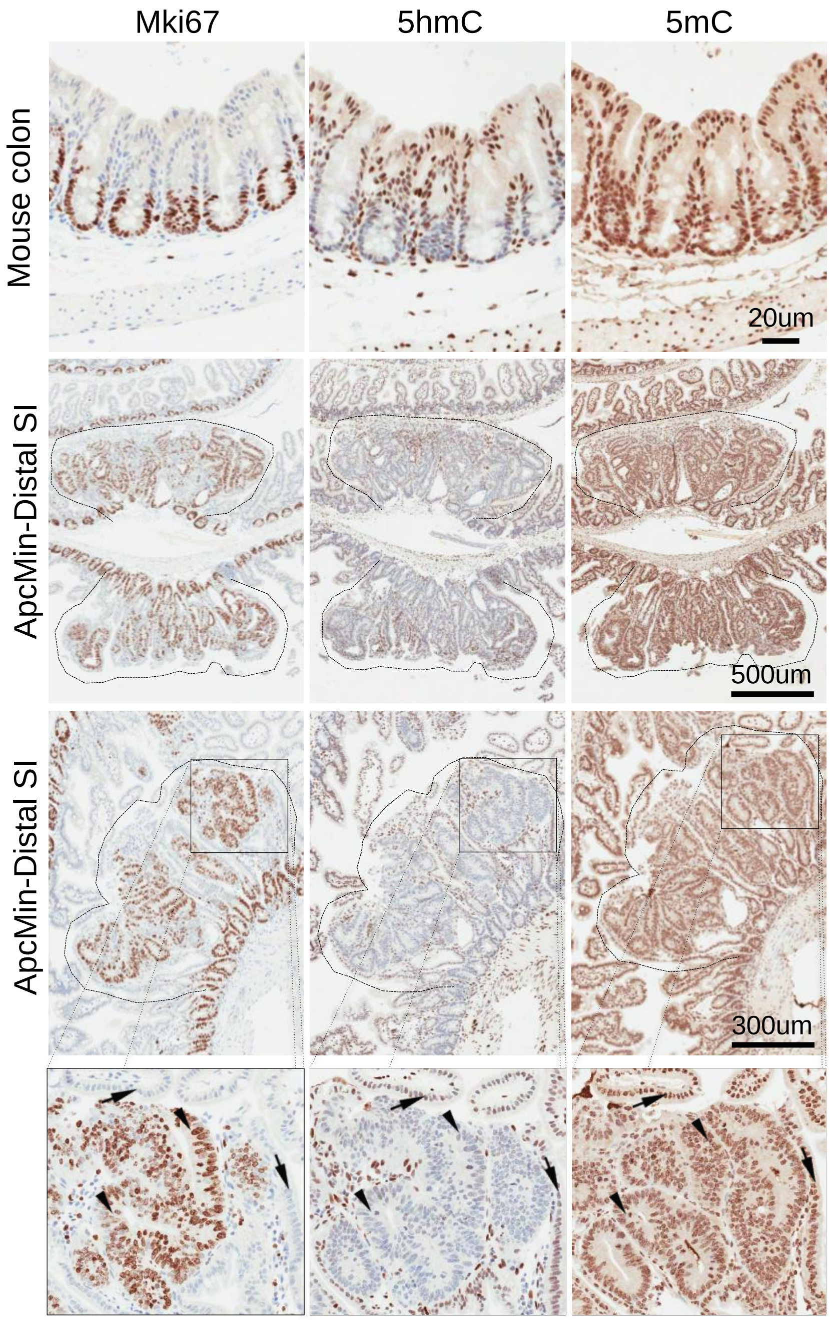

Supplementary Figure S1. Mki67, 5hmC and 5mC in the mouse colon and two examples of staining patterns in ApcMin small intestinal adenomas. Arrowheads indicate Mki67 positive cells that are negative (or low) for 5hmC but positive for 5mC . Arrows indicate Mki67 negative cells positive for 5hmC and 5mC.

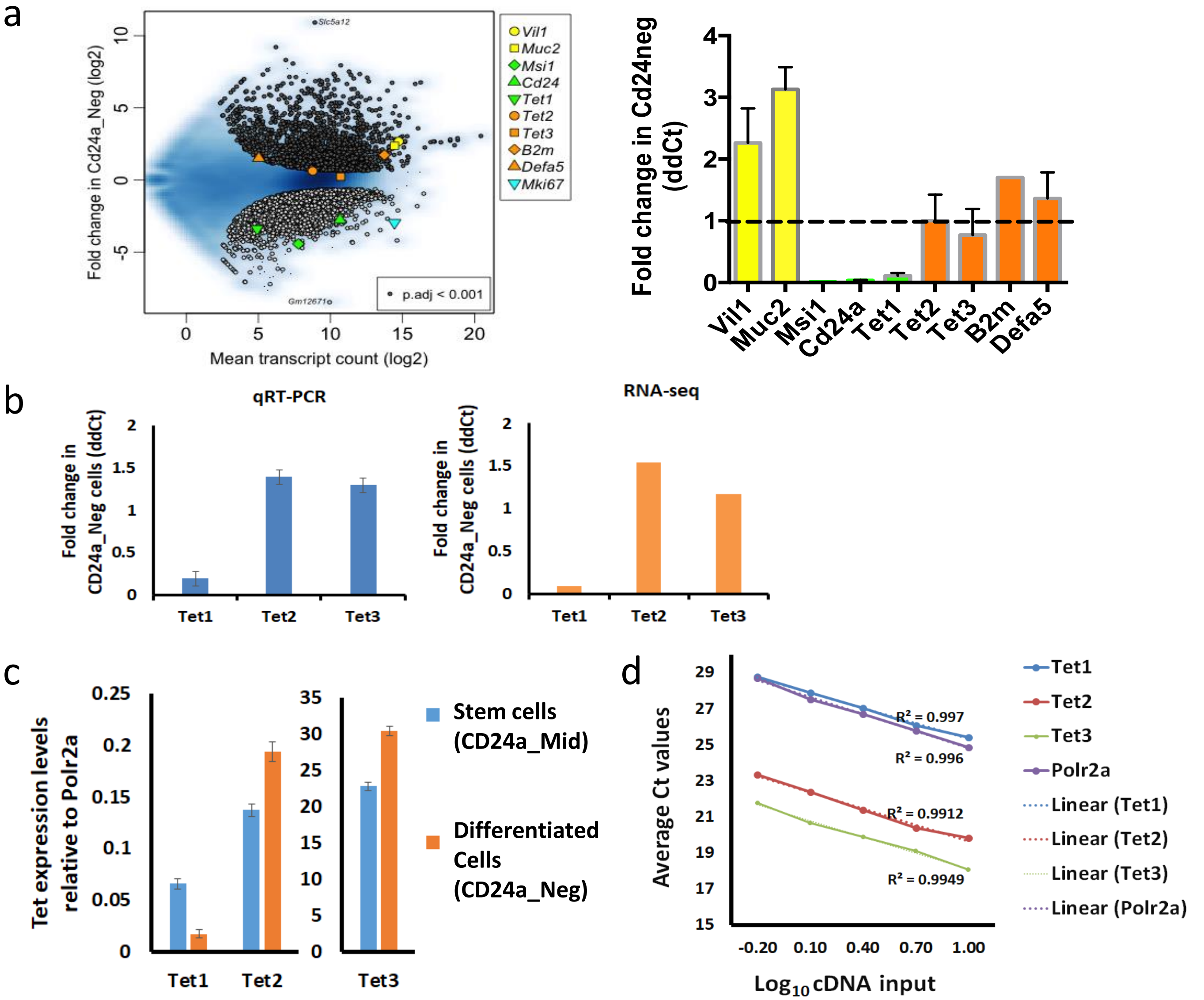

e

	log10 slope	Primer efficiency based on the log10 slope	E value (Pfaffl method)
Tet1	-2.84	125.09	2.25
Tet2	-2.99	115.77	2.16
Tet3	-2.97	116.94	2.17
Polr2a	-3.13	108.67	2.09

Supplementary Figure S2. qPCR validation of RNA-seq and specifically *Tet* transcript levels.

a. Left panel: MA plot for fold change in expression of upregulated (dark grey) or downregulated (light grey) loci in the Cd24a_Neg relative to the Cd24a_Mid progenitors. Increased expression of differentiation-specific loci *Villin1 (Vil1)* and *Mucin2 (Muc2)* is observed together with reduced expression of the stem cell marker *Musashi1 (Msi1)* and *Cd24a*. *Tet1* levels are reduced in Cd24a_Neg from an already low level of expression in the Cd24a_Mid progenitors, whereas the levels of *Tet2* and *Tet3* are maintained in the Cd24a_Neg differentiated cells. *B2m* microglobulin 'housekeeper' gene is upregulated in Cd24a_Neg. The Paneth-cell marker *Defensin alpha 5 (Defa5)* is only moderately increased in Cd24a_Neg from a low level in Cd24a_Mid as expected from Paneth-cell depletion with Ulex-lectin in the flow sort. These loci are validated by qPCR (Right panel)

b. Independent validation of Tet expression changes comparing expression changes by qRT-PCR results compared to RNA-seq results for progenitors (CD24a_Mid) and differentiated progeny (CD24a_Neg).

c. *Tet* transcript levels relative to the *Polr2a* reference gene in both the CD24a_Mid and CD24a_Neg cells showing that Tet3 is the most abundant transcript.

d. The standard curves for the primer pairs used in the above experiments depicting the linearity of amplification efficiency in a 10-fold cDNA dilution series.

e. Primer efficiencies for Tet and reference genes (percentage primer efficiency and E values for the Pfaffl method calculations) The error bars represent the standard deviation of three independent replicates

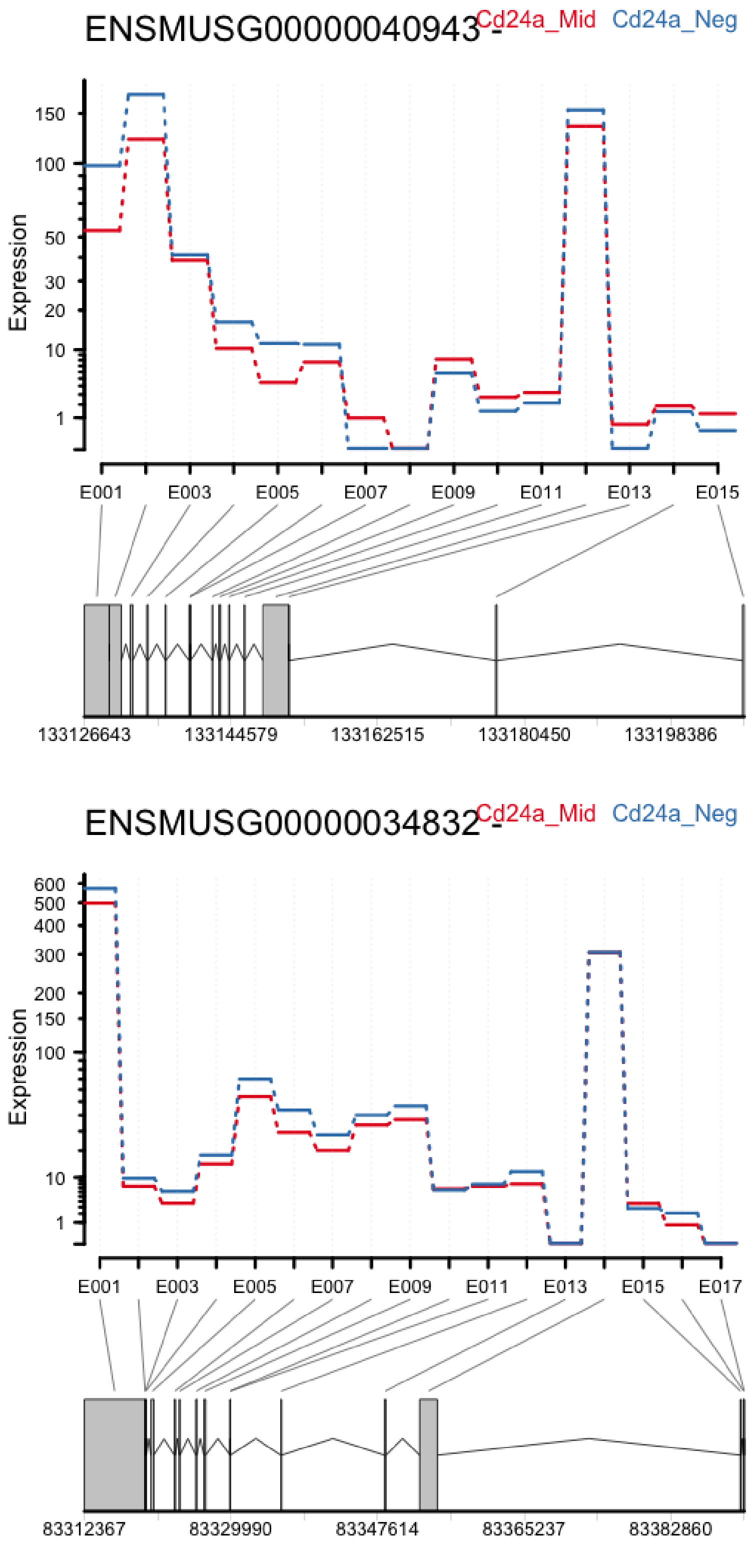

Supplementary Figure S3. Isoform analysis of *Tet2* and *Tet3* transcripts. DEXSeq plots for *Tet2* (top panel) and *Tet3* (bottom panel) in Cd24a_Mid and Neg. Statistical significance was not observed for read counts within exons between CD24a_Mid and Cd24a_Neg conditions. Alternative exon usage therefore does not appear to occur at these loci.

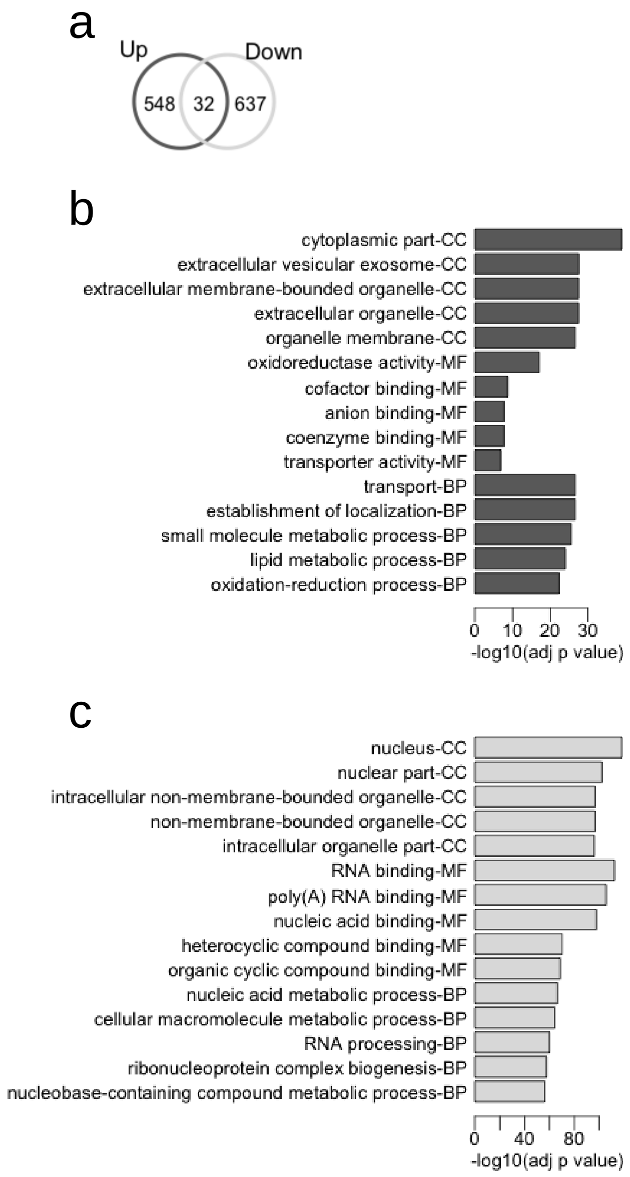

Supplementary Figure S4. RNA-seq GO analysis**.**

a. Venn diagram intersecting significantly enriched GO categories (adjusted p value < 0.05) associated with genes that were Up or Downregulated (see Additional file 2 for full analysis data).
b. Top 15 categories uniquely associated with transcriptionally upregulated loci in Cd24a_Neg relative to Cd24a_Mid. CC=cellular compartment, MF=molecular function, BP=biological process.
c. Top 15 categories uniquely associated with transcriptionally downregulated loci in Cd24a_Neg relative to Cd24a_Mid.

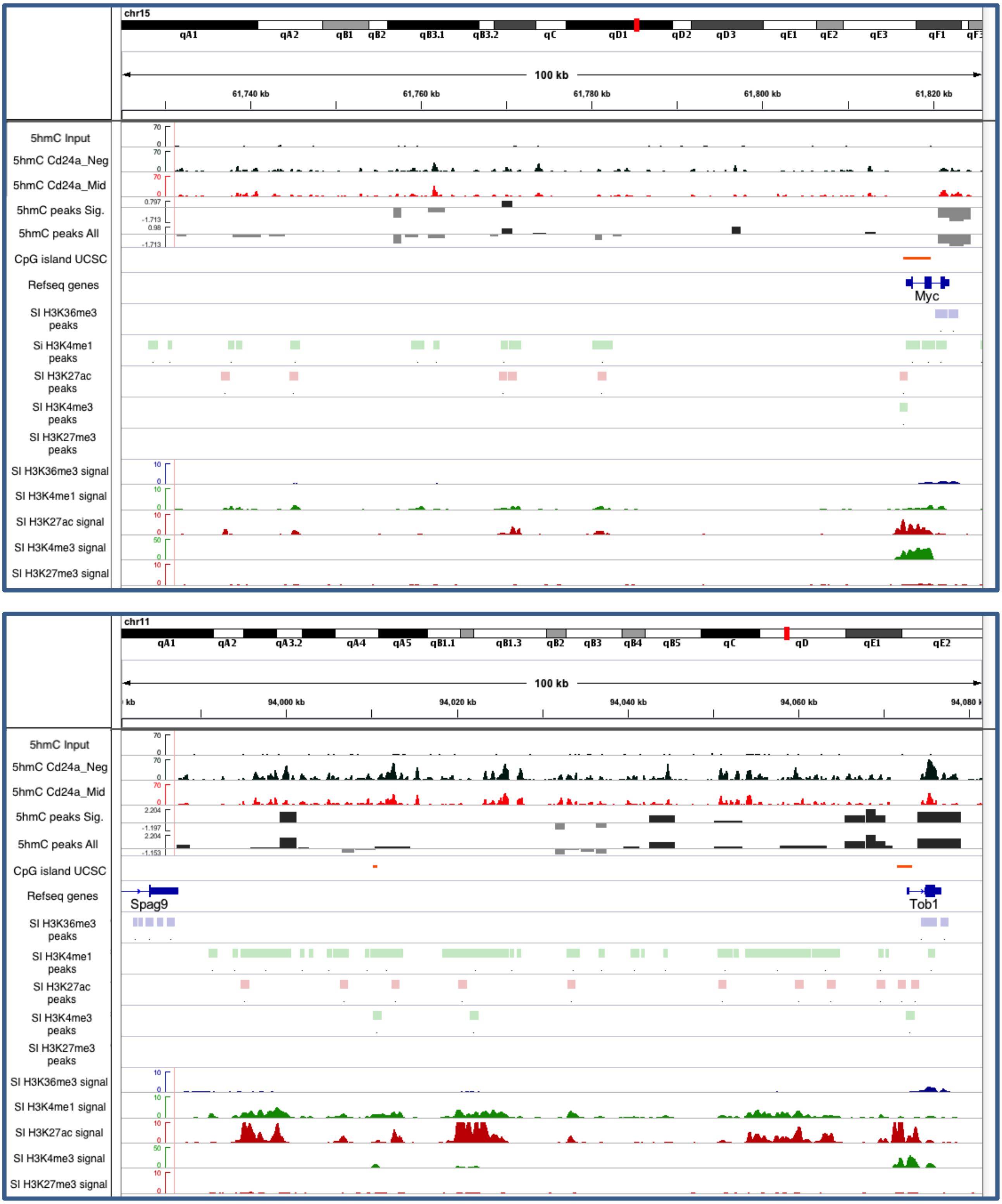

Supplementary Figure S5. IGV browser shots for upstream regions of *Myc* (top panel) and *Tob1* (bottom panel). Related to figure 3. Shown are:

1. Tracks of hmeDIP-seq bigWigs from input and bound Cd24a_Mid and Cd24a_Neg DNA. Each bigWig was generated by merging reads from inputs (one of each Cd24a population) and bound fractions (five of each Cd24a population).
2. Tracks for 5hmC peaks called from hmeDIP-seq bound fractions normalized to input DNA. Gain or loss of 5hmC in Cd24a_neg relative Cd24a_Mid are shown in dark and light grey respectively. The track height is proportional to the fold change (log2) in hmeDIP-seq read content between conditions. Sig=peaks with an adjusted p value <0.001, All=all peaks.
3. Track of CpG islands from the UCSC annotation.
4. Track of Refseq gene models.
5. Tracks for ENCODE small intestine (SI) ChIP-seq peaks and wig signals of the indicated histone modifications.

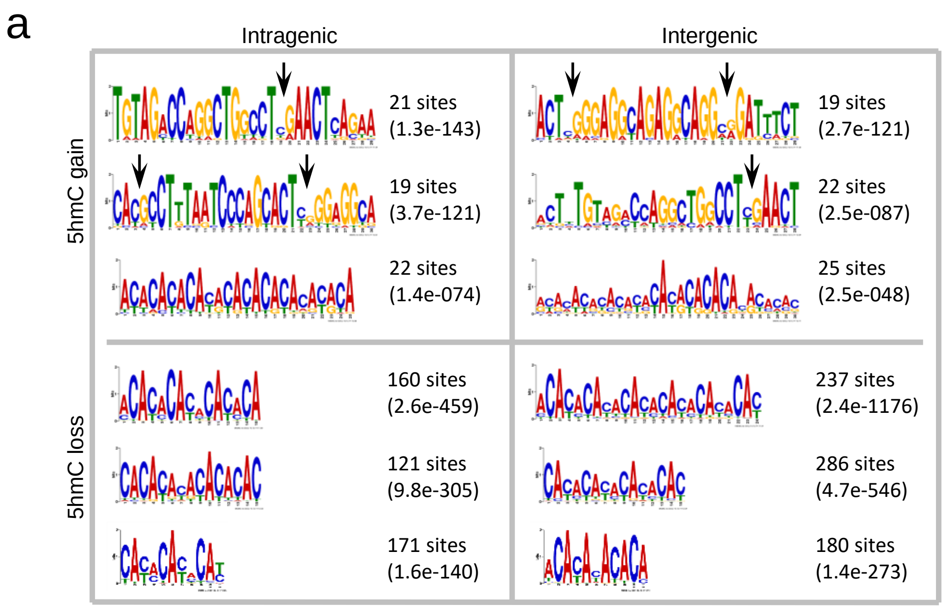

b

	N of 5hmC peaks with (CA)n repeats	% of 5hmC All (93709 peaks)	% of 5hmC Gain (21858 peaks)	% of 5hmC Loss (21567 peaks)
All	22209	22.8	–	–
Gain	3611	3.7	16.5	–
Gain intragenic	2678	2.8	12.3	–
Gain intergenic	933	1	4.3	–
Loss	8125	8.3	–	37.7
Loss intragenic	3694	3.8	–	17.1
Loss intergenic	4431	4.6	–	20.5

Supplementary Figure S6. 5hmC peaks motif analysis (MEME-ChIP) and (CA)n repeat overlaps (bedtools).

a. Motifs found for intragenic and intergenic peaks <= to 1kb in length and with a significant change (adjusted p value <0.001) in 5hmC in Cd24a_Neg progeny relative to the Cd24a_Mid progenitors. 1507 and 725 sequences were submitted for intragenic and intergenic gain respectively and 2354 and 2830 sequences for intragenic and intergenic loss respectively. The number of sites and (E-value) are indicated. Arrows indicate presence of CpG dinucleotides.

b. Summary table for the number (N) of 5hmC peaks that fully contain (CA)n simple repeats. From 93709 5hmC peaks identified 22209 (22.9%) contained repeats. The breakdown of Inter- and intragenic gain and loss within this 22% is as shown. The percentage of peaks with repeats for 5hmC gain is 3611/21858 (16.5%) and for loss is 8125/21567 (37.7).

a

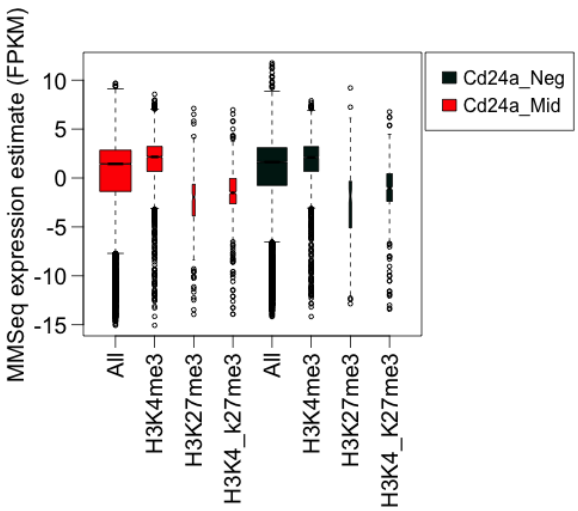

b

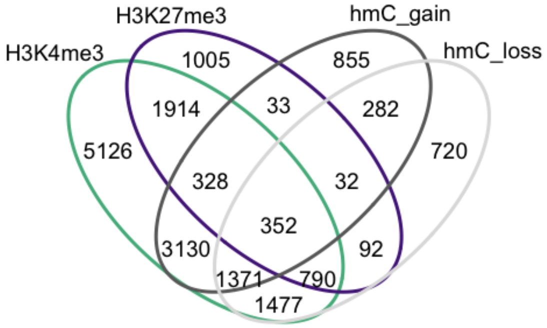

Supplementary Figure S7. Expression levels and intragenic 5hmC change of genes with H3K4me3 and/or H3K27me3 peaks within 5kb of the TSS.

a. Expression levels of genes with promoters marked by H3K4 and/or H3K27me3 (ENCODE ChIP-seq) in Cd24a_Mid and Neg cells. The width of the boxes is proportional to the numbers of promoters. The ENCODE ChIP-seq peaks were annotated to the TSS with PeakAnalyzer (PMCID: PMC2923140).

b. Venn diagram intersecting symbols of genes marked by H3K4me3 and/or H3K27me3 in the promoter and the change in 5hmC in the gene body (p.adj < 0.001). Genes with H3K4me3 monovalent promoters more frequently gained 5hmC (3130 vs 1477) whereas genes with bivalent H3K4/K27me3 or monovlalent H3K27me3 promoters more frequently lost 5hmC (790 vs 328 and 92 vs 33 respectively). Note that a number of genes gained and lost 5hmC in the gene body (see main Fig.4) and that 5hmC is very rare at genes with monovalent H3K27me3 promoters.

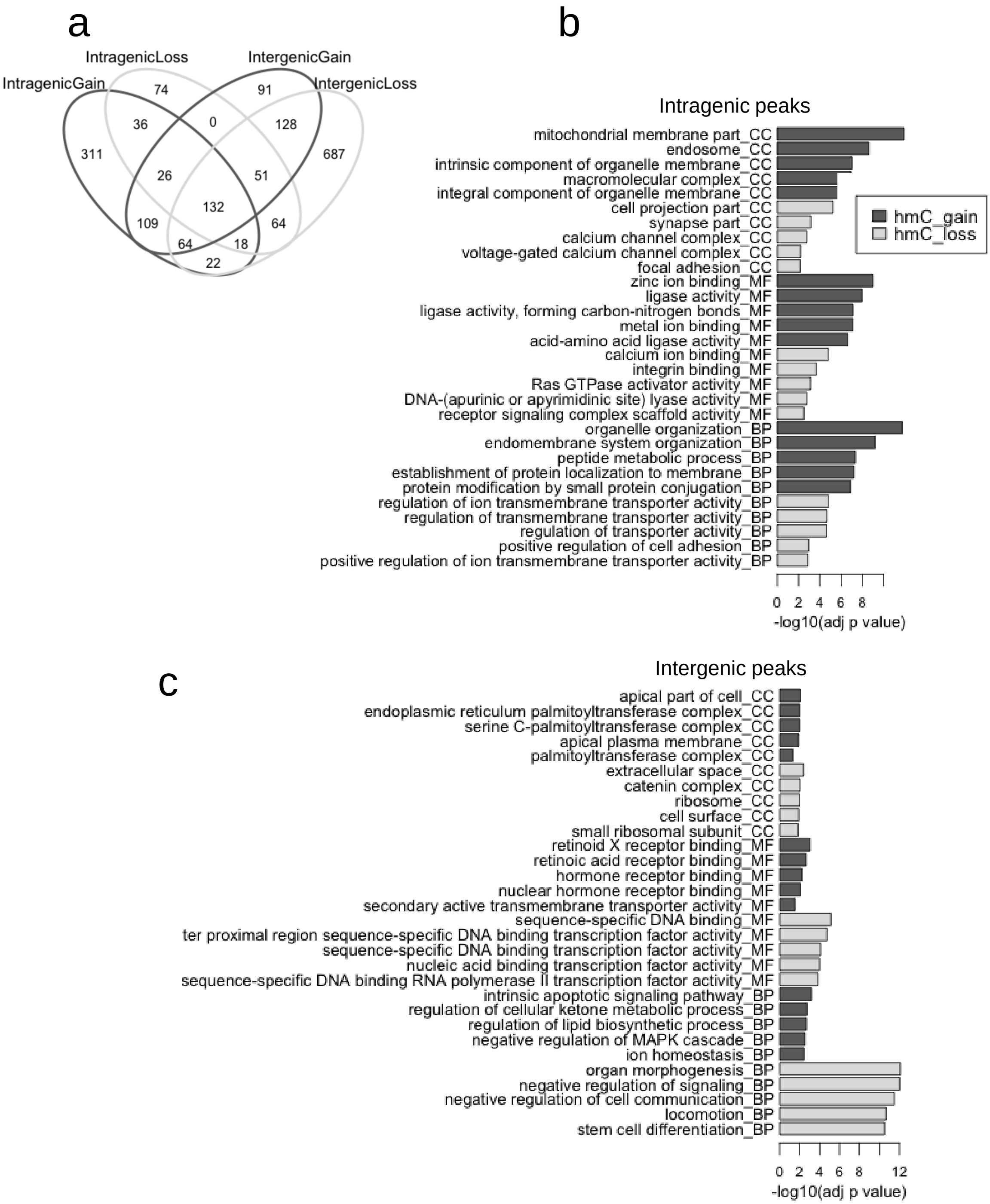

Supplementary Figure S8. hmeDIP-seq GO analysis.
a. Venn diagram intersecting significantly enriched GO categories (p.adj < 0.05) to identify those that are unique to each condition analysis (see Additional file 2 for full analysis data). **b.** Top 15 GO categories from those uniquely associated to genes with intragenic peaks that gain 5hmC (311 categories) or lose 5hmC (74 categories). **c.** Top 15 GO categories from those uniquely associated to genes with 'cis-acting' intergenic peaks that gain 5hmC (91 categories) or lose 5hmC (687 categories). CC = cellular compartment, MF = molecular function, BP = biological process. See additional file 2 for full GO analysis data.

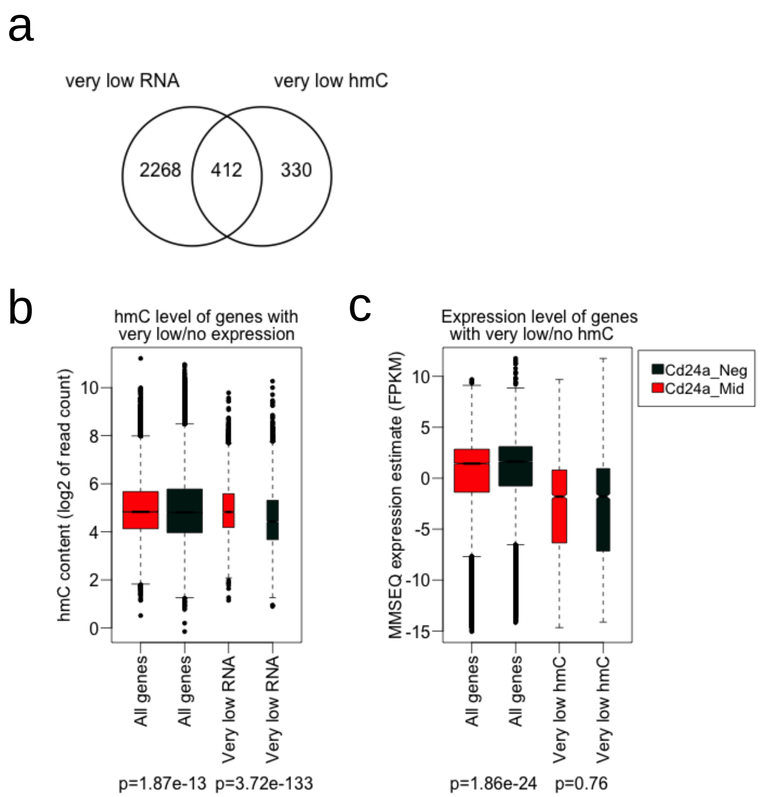

Supplementary Figure S9. 5hmC was present at constitutively repressed genes and reduced in differentiation.

a. Venn diagram intersecting genes where RNA or 5hmC were undetected (very low).
b. Boxplot for 5hmC content in all genes and in genes with undetected RNA only (2258 genes) in Cd24a_Mid and Neg cells. The reduction of 5hmC upon differentiation occurred at intragenic and intergenic sites (40% and 60% respectively out of 4612 peaks, not shown).
c. Boxplot for RNA levels (MMseq expression estimates) in all genes and in genes with undetected 5hmC only (330 genes) in Cd24a_Mid and Neg cells. P values were obtained by a Wilcox test.

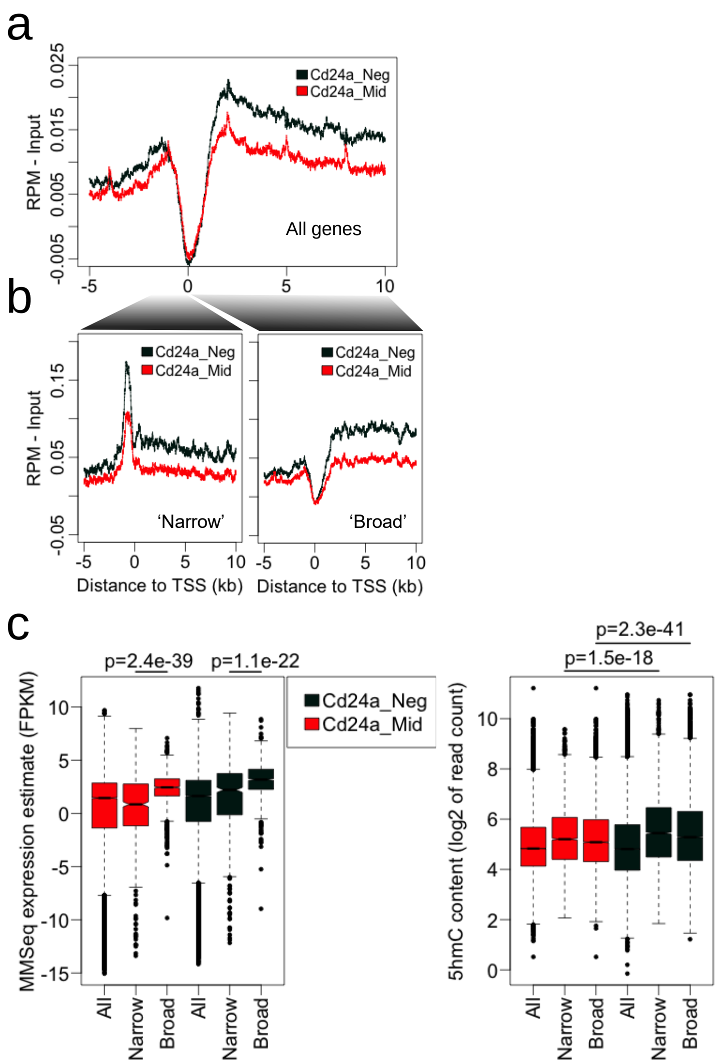

Supplementary Figure S10 The content of 5hmC around the TSS and its correlation with expression levels.

a. hmeDIP-seq profile for all genes around the TSS in the Cd24a_Mid and Neg cells. Reads Per Million mapped reads minus the input (RPM – Input).

b. Left – the top 1000 genes ranked by 5hmC content in a 1kb window upstream of the TSS show a 'Narrow' 5hmC profile. Right – the top 1000 genes ranked by 5hmC content in the gene body (from the TSS to the TTS) show a 'Broad' 5hmC profile.

c. Left panel – expression levels of genes associated with the Narrow and Broad profiles. The MMSeq expression estimate is in Fragments per Kb of transcript per Million mapped reads (FPKM). Genes with a higher content of 5hmC near the TSS (Narrow type) are more variable, and show a significantly lower level of expression in the Cd24a_Mid and Neg cells relative to the Broad type (Wilcox test). However both Narrow and Broad promoter genes are for the most part active loci in both Mid and Neg cells and gain expression and 5hmC (right panel) in Cd24a_Neg cells.

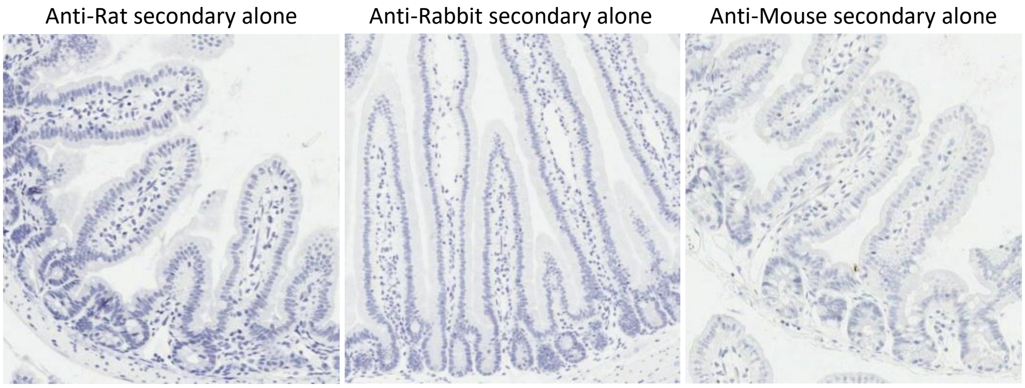

Supplementary figure S11. Mouse small intestine secondary antibody alone IHC stains. The anti-Rat, anti-Rabbit, and anti-Mouse secondaries stains are the no primary antibody controls for signals obtained with Rat-anti-Mki67, Rabbit-anti-5hmC, and Mouse-anti-5mC, respectively.
